# Crystal structure and Hirshfeld surface analysis of tri­chlorido­(1,10-phenanthroline-κ^2^*N*,*N*′)phenyltin(IV)

**DOI:** 10.1107/S2056989024009150

**Published:** 2024-09-24

**Authors:** Tarek Benlatreche, Mohamed Abdellatif Bensegueni, Georges Dénès, Stéphane Golhen, Hocine Merazig

**Affiliations:** a Environmental and Structural Molecular Chemistry Research Unit URCHEMS Faculty of Exact Sciences University of Constantine 1-Mentouri Brothers 25000 Algeria; b National Higher School for Hydraulics, Abdellah Arbaoui, Blida, Algeria; c Laboratory of Solid State Chemistry and Mössbauer Spectroscopy, Chemistry and Biochemistry Department, Concordia University, Montreal, Canada; d CNRS, Rennes Institute of Chemical Sciences – UMR 6226, University of Rennes, France; Venezuelan Institute of Scientific Research, Venezuela

**Keywords:** crystal structure, Hirshfeld surface analysis, C—H⋯Cl hydrogen bond, phenanthroline, tin(IV)

## Abstract

The title compound, [Sn(C_6_H_5_)Cl_3_(C_12_H_8_N_2_)], which was obtained by the reaction between 1,10-phenanthroline and phenyl­tin trichloride in methanol, exhibits intra­molecular hydrogen-bonding inter­actions involving the chlorine and hydrogen atoms. Crystal cohesion is ensured by inter­molecular C—H⋯Cl hydrogen bonds, as well as *Y*—*X*⋯π and π-stacking inter­actions involving three different aromatic rings with centroid–centroid distances of 3.6605 (13), 3.9327 (14) and 3.6938 (12) Å]. Hirshfeld surface analysis and the associated two-dimensional fingerprint plots reveal significant contributions from H⋯H (30.7%), Cl⋯H/H⋯Cl (32.4%), and C⋯H/H⋯C (24.0%) contacts to the crystal packing while the C⋯C (6.2%), C⋯Cl/Cl⋯C (4.1%), and N⋯H/H⋯N (1.7%) inter­actions make smaller contributions.

## Abstract 2

The title compound, which was obtained by the reaction between 1,10-phenanthroline and phenyl­tin trichloride in methanol, exhibits intra­molecular inter­actions involving the chlorine and hydrogen atoms. Crystal cohesion is ensured by inter­molecular C—H⋯Cl hydrogen bonds, as well as *Y*—*X*⋯π and π-stacking inter­actions

## 1. Chemical context

Complexes of 1,10-phenanthroline (Phen) with *d*-metals have attracted much inter­est because of the adaptability and chemical properties of Phen (Sammes & Yahioglu, 1994 ▸), that confers additional properties upon coordination with other metals and thus opens up new areas of investigation. Tin(IV) complexes are widespread in chemistry and play a significant role in biology, industry, and agriculture (Syed Annuar *et al.*, 2021 ▸; Ross, 2006 ▸) as theis class of compounds has shown efficacy against a wide range of diseases and they have strong biological activities such as anti­fungal (Rebolledo *et al.*, 2003 ▸), anti­bacterial (Al-Allaf *et al.*, 2003 ▸), anti-proliferative and anti­tumor (Banti *et al.*, 2019 ▸) properties.
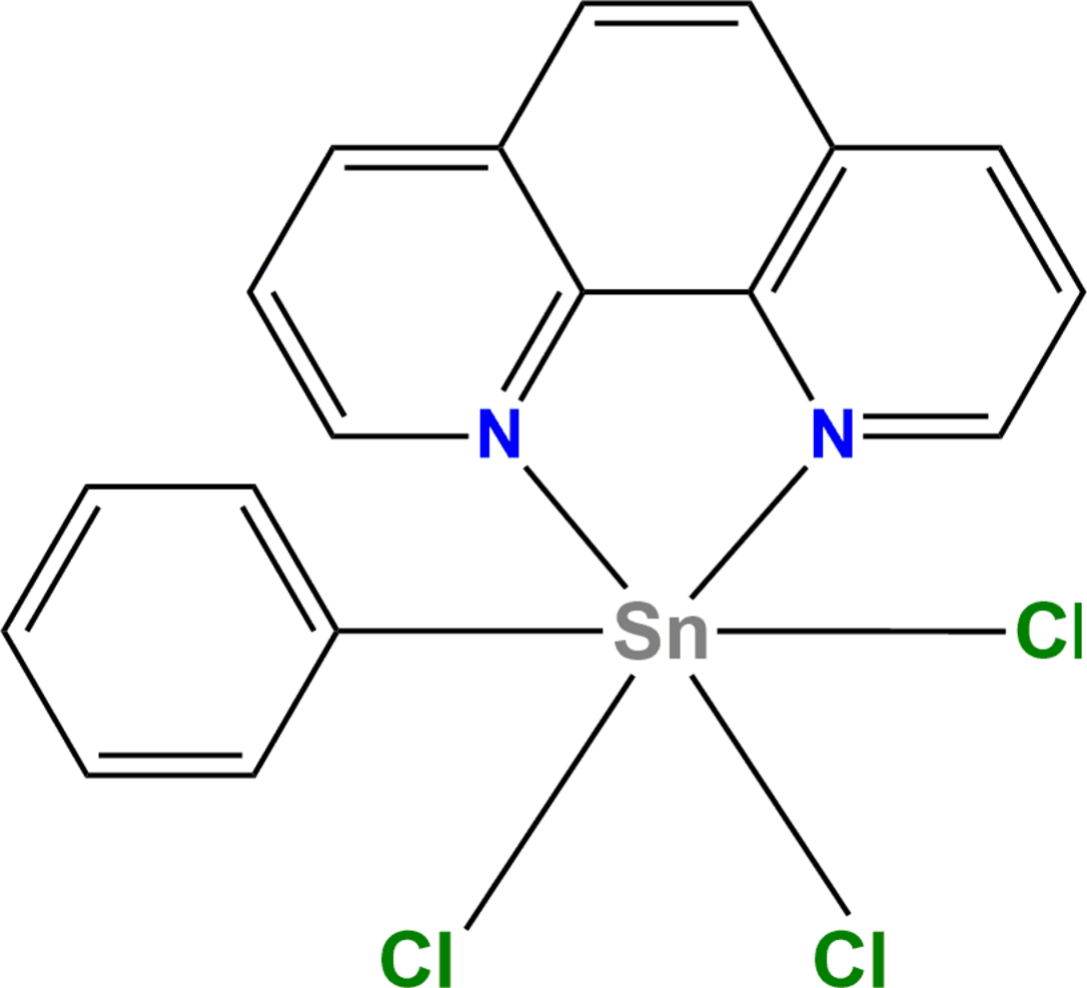


The synthesis of the title compound along with the crystal structure and spectroscopic characterization, as well the results of a Hirshfeld surface analysis are all reported here.

## 2. Structural commentary

**Figure 1:**
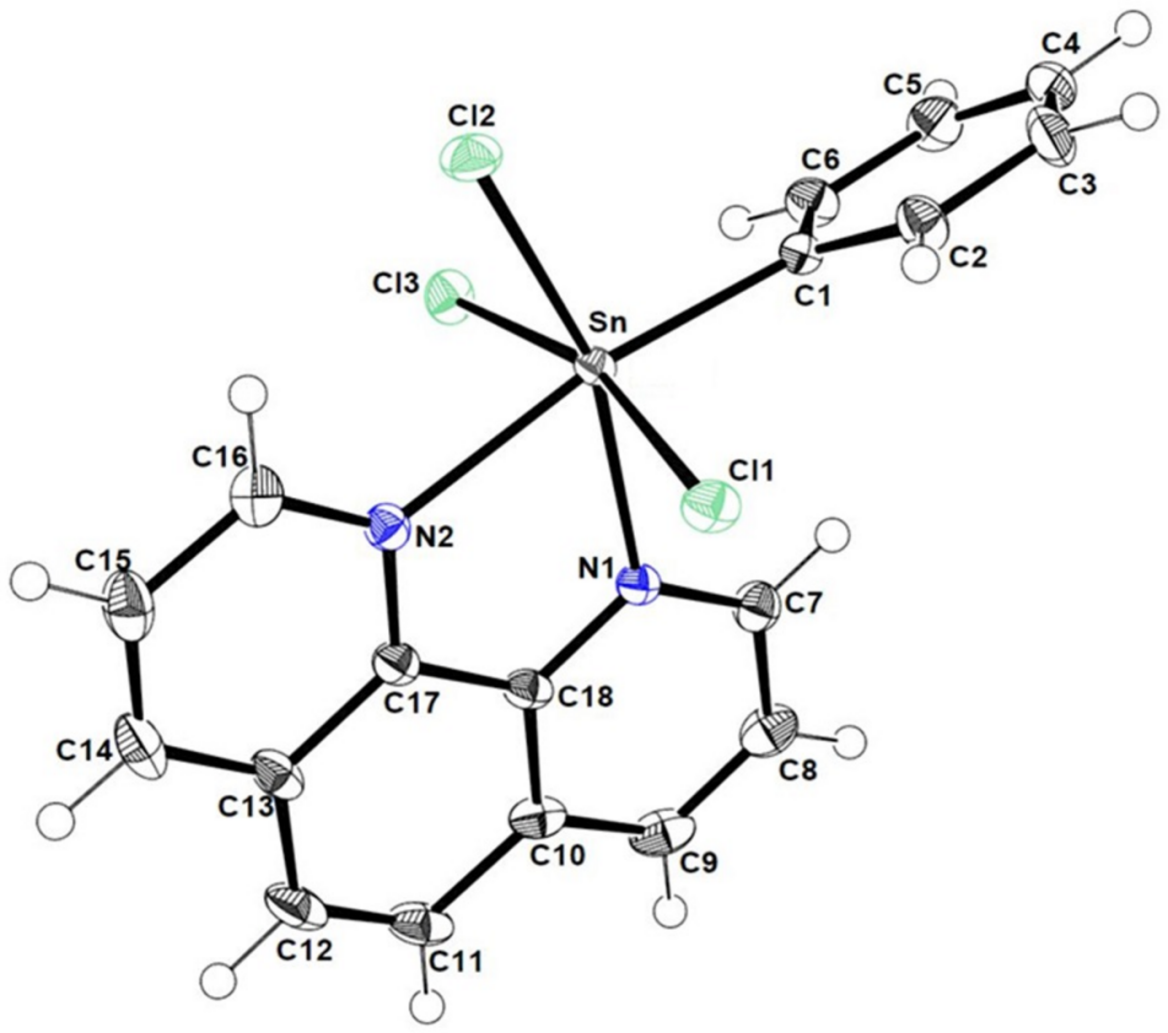
The asymmetric unit of the title compound with displacement ellipsoids drawn at the 50% probability level. H atoms are represented as small circles.

**Figure 2:**
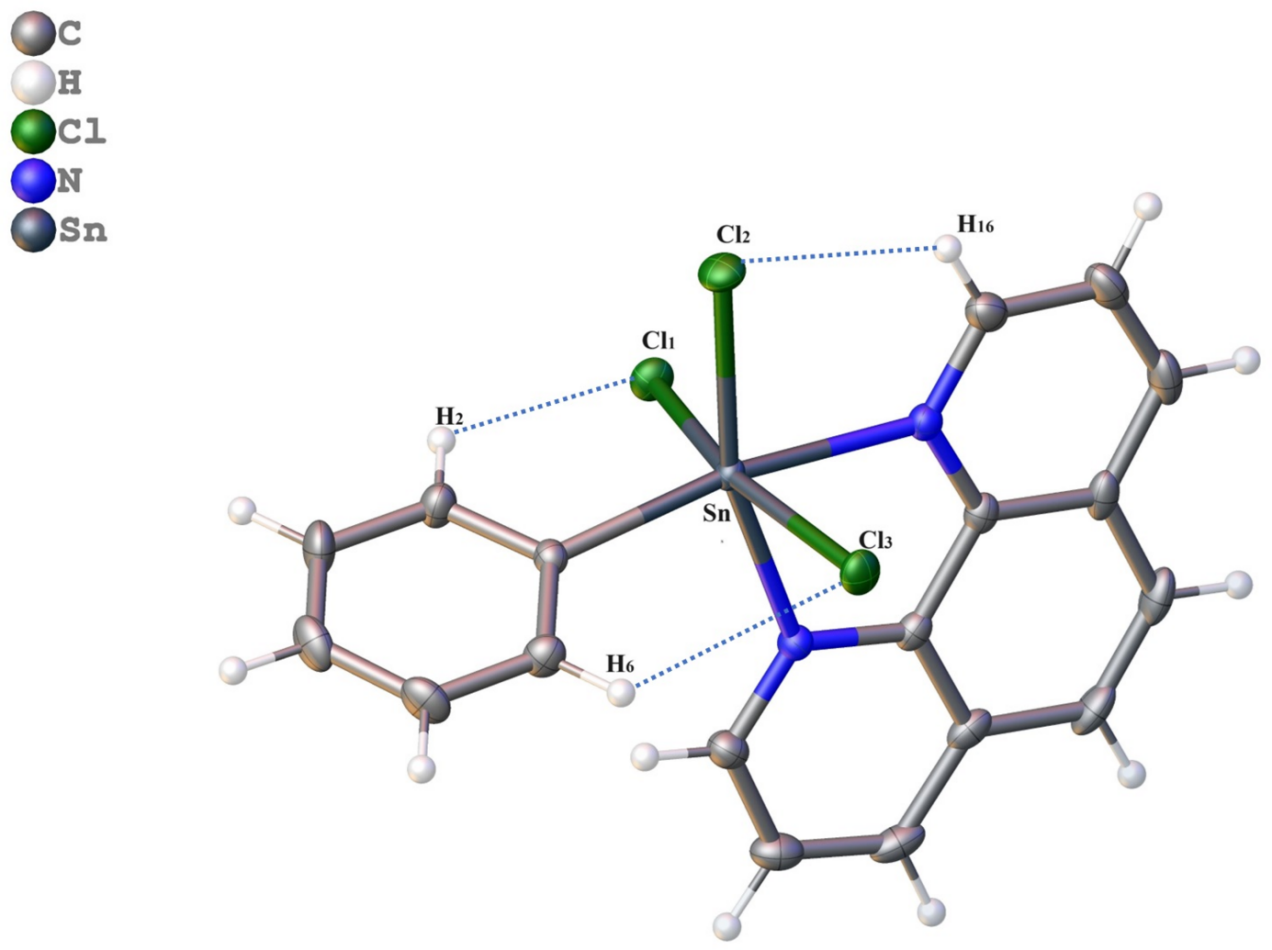
Intra­molecular hydrogen bonds directing the conformation of the structure.

The title complex (Fig. 1 ▸) crystallizes in the monoclinic space group *P*2_1_/*n*. Bond lengths and angles are comparable with those previously reported for related structures (Hall *et al.*, 1996 ▸). The tin atom is six-coordinate, being chelated by two nitro­gen atoms (N1 and N2) of the 1,10-phenanthroline ligand and coordinated by a carbon atom of the phenyl ligand (C1), and three chlorine atoms (Table 1 ▸). The geometry of the tin atom is distorted octa­hedral with angles ranging from 72.77 (7) to 168.92 (8)°, the smallest being between the tin atom and the two nitro­gen atoms and the largest is between the tin and carbon atom of the phenyl and the nitro­gen atom of the ligand. The dihedral angle between the planes through the phenyl ring and the phenanthroline ligand is 69.73 (9)°. Intra­molecular C—H⋯Cl hydrogen bonds are observed (Table 2 ▸), characterized by *D*⋯*A* distances of 2.75, 2.86 and 2.97 Å. These interactions play a vital role in maintaining the specific conformation of the mol­ecule, thus enhancing its overall rigidity (Fig. 2 ▸).

## 3. Supra­molecular features

The crystal structure is intricately organized, primarily upheld by weak inter­molecular C—H⋯Cl hydrogen bonds (Table 3 ▸), *Y*—*X*⋯π and π-stacking inter­actions. These inter­actions act as the framework for structural cohesion, effectively connecting individual mol­ecules.

**Figure 3:**
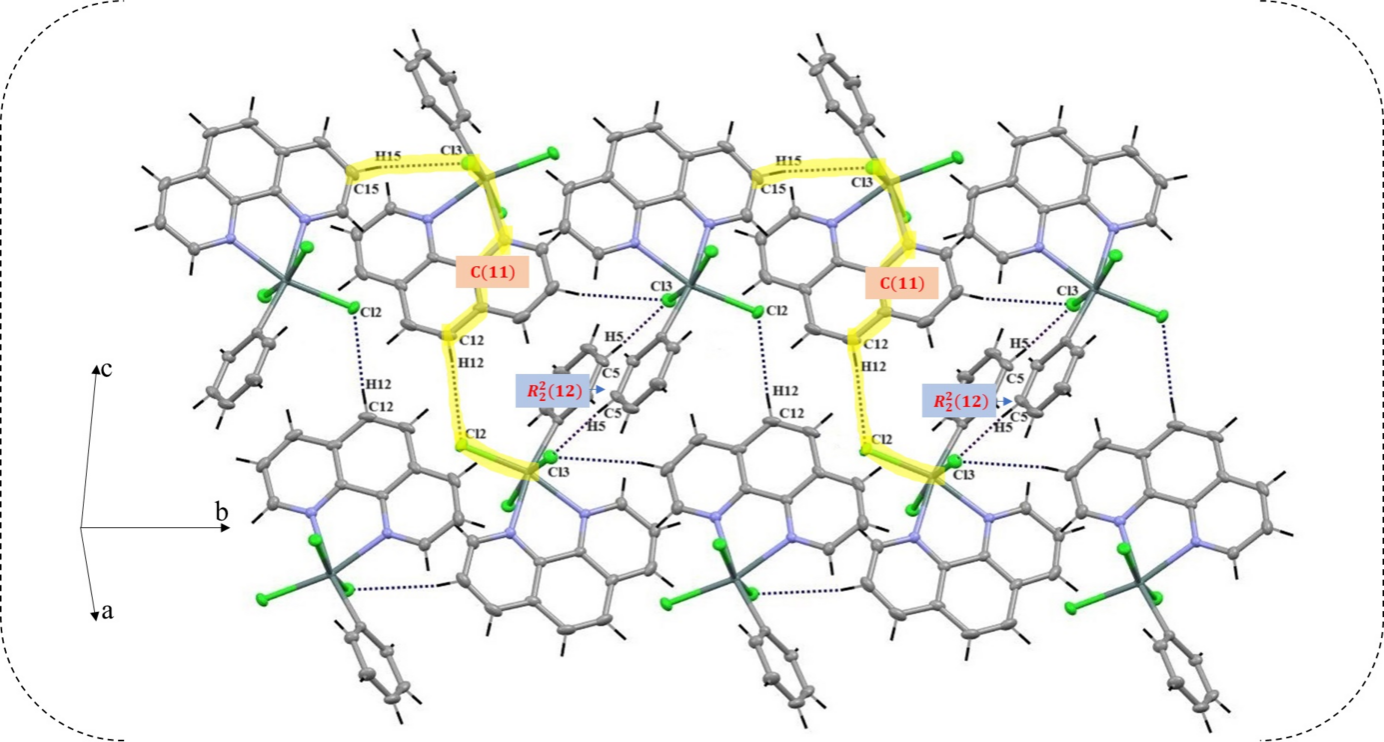
Inter­molecular hydrogen bonds: C15—H15⋯Cl3^i^, C5—H5⋯Cl3^ii^, and C12—H12⋯Cl2^v^ and hydrogen-bonded planes in the title compound. [Symmetry codes: (i) −*x* + 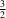, *y* + 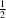, −*z* + 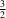; (ii) −*x* + 1, −*y* + 1, −*z* + 2; (v) *x* + 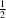, −*y* + 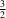, *z* − 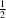.]

**Figure 4:**
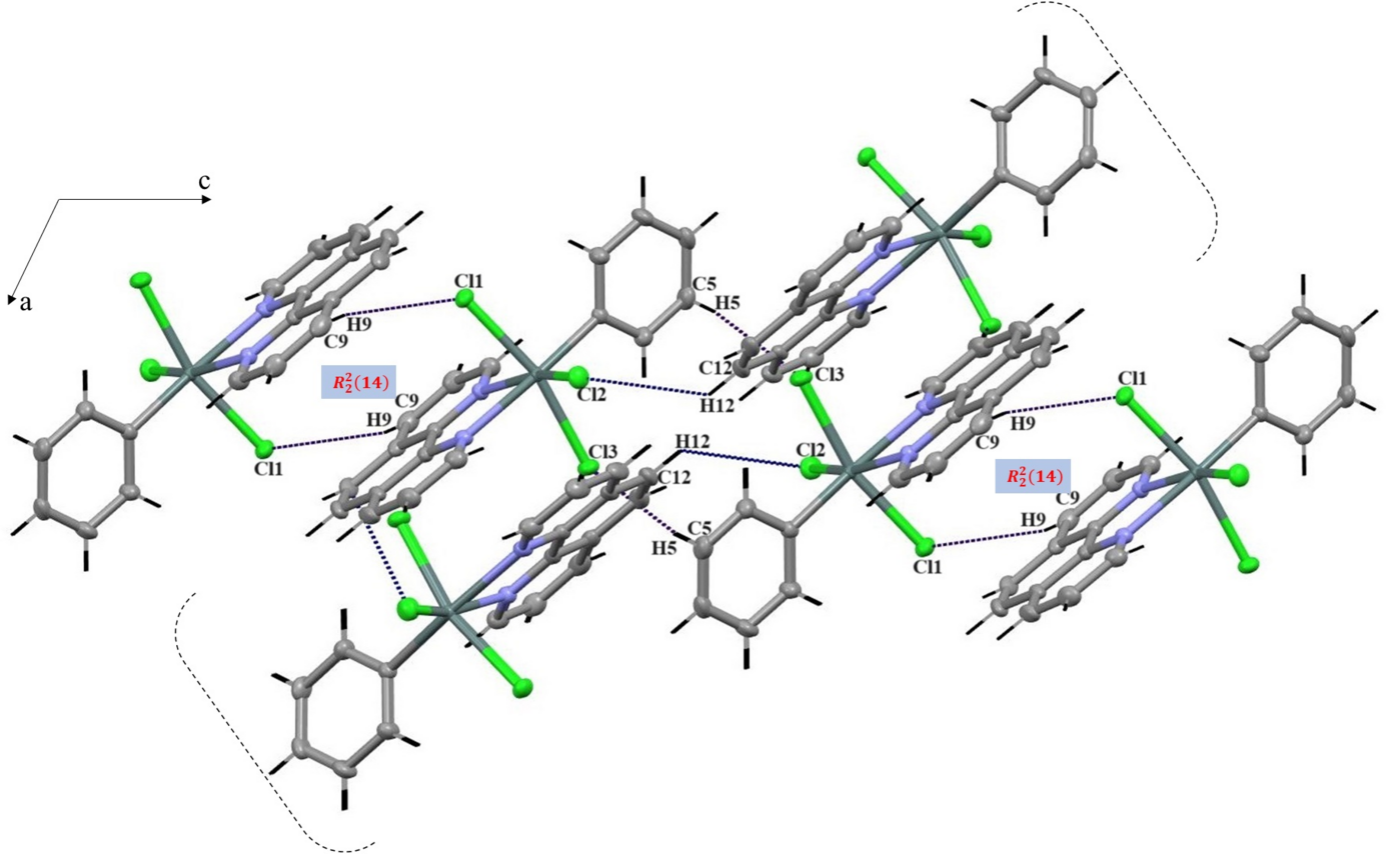
Linkage of planes along the *c* axis by C9—H9⋯Cl1^iii^ hydrogen bonds, forming 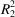
(14) rings. [Symmetry code: (iii) −*x* + 1, −*y* + 1, −*z* + 1.]

Within this framework, the inter­molecular C15—H15⋯Cl3, C5—H5⋯Cl3 and C12—H12⋯Cl2 hydrogen bonds, with the H⋯*A* distances of 2.94, 2.84 and 2.97 Å, respectively, create bridges between adjacent mol­ecules. These hydrogen bonds generate rings with an 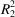
(12) motif and *C*(11) chains (Etter *et al.*, 1990 ▸), which align along the *b*-axis direction, creating hydrogen-bonded planes parallel to the *ab* plane (Fig. 3 ▸) (Etter *et al.*, 1990 ▸). These planes, in turn, are linked along the *c*-axis by C9—H9⋯Cl1 hydrogen bonds generating 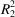
(14) hydrogen-bonded rings (Fig. 4 ▸); this bonding mechanism facilitates cohesion and contributes to the consolidation of the crystal structure.

**Figure 5:**
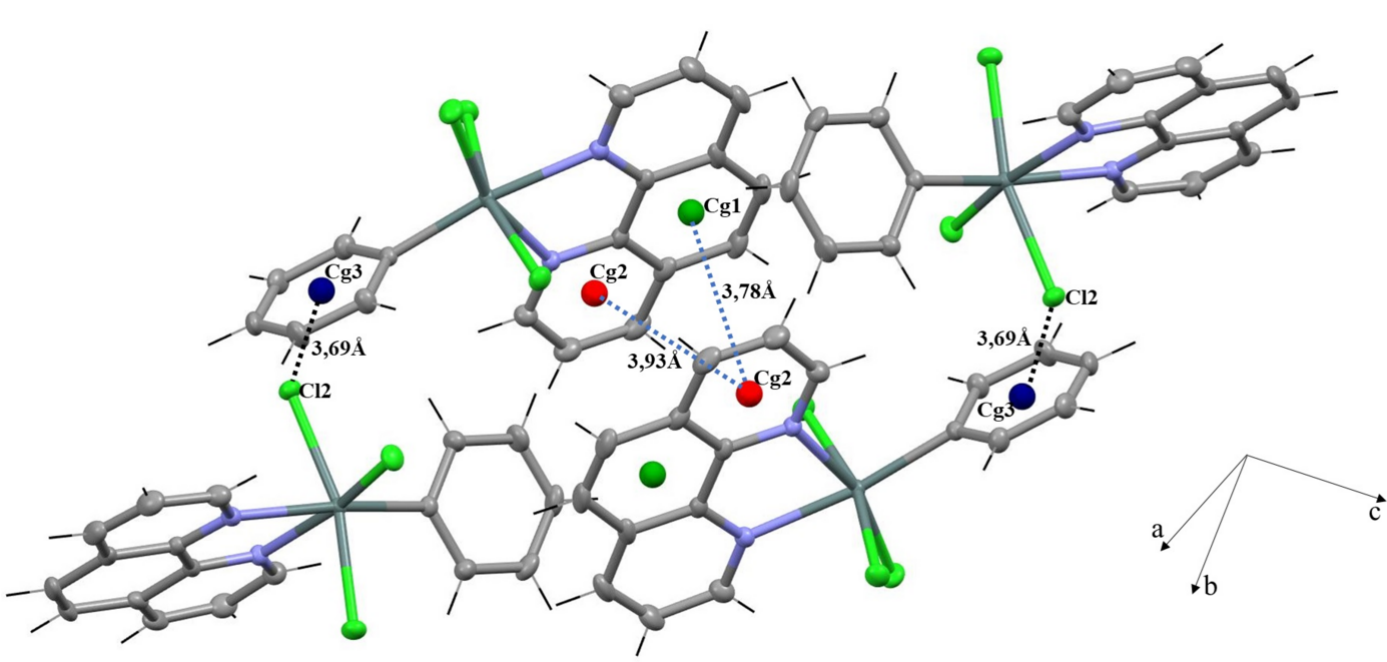
π-stacking and Y—*X*⋯π inter­actions between 1,10-phenanthroline rings, reinforcing the structure.

The three-dimensional architecture is further consolidated by π-stacking inter­actions between 1,10-phenanthroline units, with centroid–centroid distances *Cg*2⋯*Cg*2(1 − *x*, 1 − *y*, 1 − *z*) = 3.9327 (14) Å and *Cg*1⋯*Cg*2(1 − *x*, 1 − *y*, 1 − *z*) = 3.6605 (13) Å where *Cg*2 and *Cg*1 are the centroids of the N1/C7–C10/C18 and C10–C13/C17/C18 rings, respectively. Additionally, *Y*—*X*⋯π inter­actions, Sn1—Cl2⋯*Cg*3(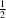 − *x*, 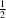 + *y*, 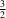 − *z*), where *Cg*3 is the centroid of the C1–C6 ring, with a Cl⋯*Cg* distance of 3.6938 (12) Å, create extra connections within the crystal (Fig. 5 ▸).

Remarkably, despite the intricate network of inter­actions, no classical hydrogen bonds or voids are detected within the structure, underscoring the efficiency of the aforementioned mechanisms in maintaining structural cohesion.

## 4. Database survey

A search of the Cambridge structural Database (CSD, version 2024.2.0, update of September 2024; Groom *et al.*, 2016 ▸) for similar compounds was undertaken. The compound CEXMIC (Su *et al.*, 2007 ▸) crystallizes with the same arrangement, differing only in the substitution of the phenyl ligand with a chloro substituent. This is also observed in TECMUJ (Hall & Tiekink, 1996 ▸), but with a different arrangement in the *P*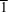 space group of the triclinic crystal system. Similarly, in ARAWOF (Casas *et al.*, 2003 ▸), with space group *P*2_1_/*n*, an ethyl group replaces the Cl atom in the coordination sphere while maintaining the same crystalline structure. CIHQUI (Klösener *et al.*, 2018 ▸) crystallizes with an identical crystal structure but exhibits halogen inter­actions and hydrogen bonding with a fluorine atom as the generator atom. AYAFEL (Ma *et al.*, 2004 ▸) crystallizes with space group *Pca*2_1_, featuring two chelations, one with the same ligand and another with a sulfur ligand, while the chloro substituents are substituted with methyls. Compound BOVHUQ (Tan *et al.*, 2009 ▸) crystallizes in the same space group, with both chloro and phenyl ligands substituted with halogenated ligands. CASVOH (Ganis *et al.*, 1983 ▸), in ortho­rhom­bic space group *P*2_1_2_1_2_1_, features chloro and phenyl ligands substituted with *n*-butyl. Similarly, in DUKTAH (Lo *et al.*, 2020 ▸), the substitution ligand is 4-chloro­phenyl. In EDUNEY (Najafi *et al.*, 2012 ▸) the chloro ligands are replaced by methyl and SCN ligands. FEDYIW (Archer *et al.*, 1987 ▸) exhibits a coordination of 4. RORMIU (Lange *et al.*, 1997 ▸) is a polymeric compound while SIZBIO (Najafi *et al.*, 2014 ▸), NEMTAB (Davis *et al.*, 2006 ▸), POYZAE (Kircher *et al.*, 1998 ▸) and TECMUJ (Hall *et al.*, 1996 ▸) include organic co-crystals in their crystal structures. Similar structures are observed for PAPTOS, PAPTUY, PAPVAG, and PAPVEK (Mo *et al.*, 2017 ▸), but with different halogen–halogen inter­actions.

## 5. Hirshfeld surface analysis

To investigate the nature of inter­molecular inter­actions and their importance in the crystal packing, a Hirshfeld surface (HS) analysis was undertaken and associated two-dimensional fingerprint plots (FP) (Spackman & Jayatilaka, 2009 ▸) were generated using *Crystal Explorer 21.5* (Turner *et al.*, 2021 ▸). The Hirshfeld surfaces were generated with high (standard) surface resolution and the 3-D *d*_norm_ surfaces were mapped using a fixed color scale ranging from 0.76 (red) to 2.4 (blue) from −0.0947 to 1.3214 Å. The 2D fingerprint plots were displayed using the expanded 1.0–2.8 Å view with distance scales *d*_e_ and *d*_i_ depicted on the graph axes.

**Figure 6:**
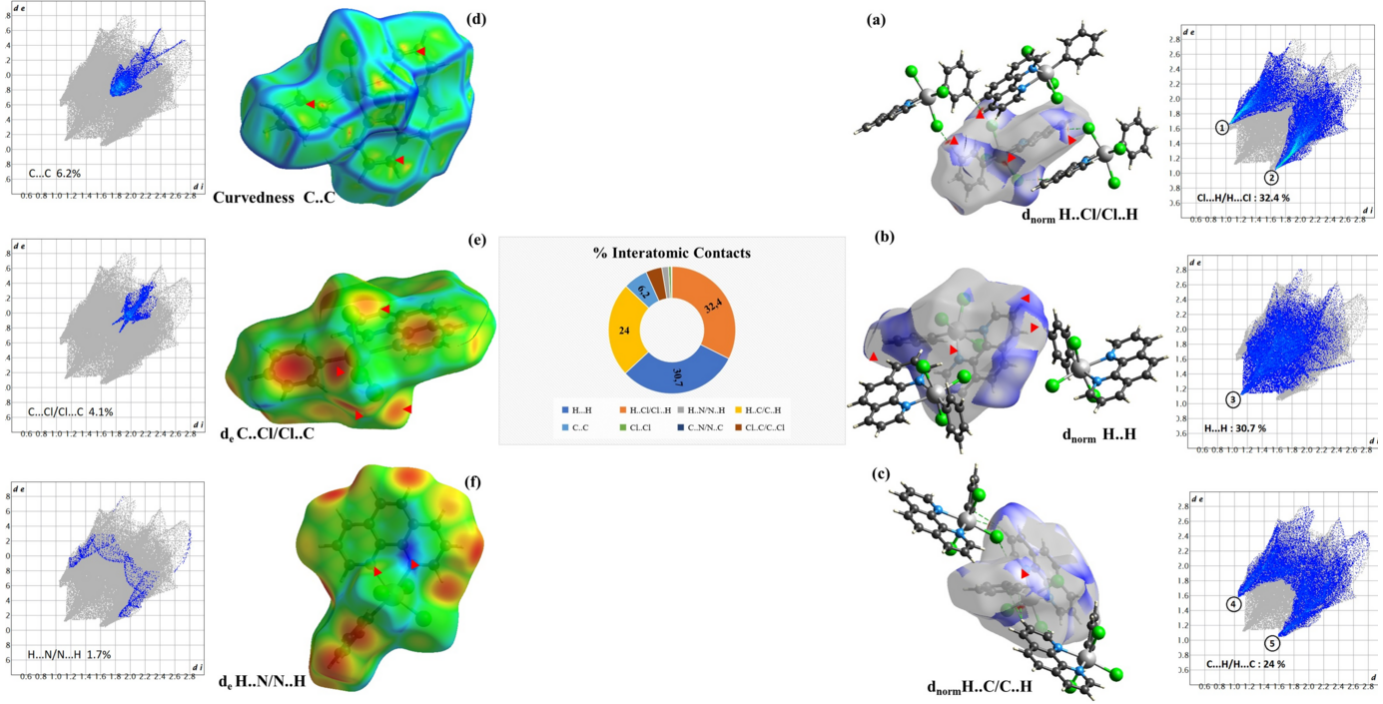
Hirshfeld surface analysis and two-dimensional fingerprints. (*a*) Hirshfeld surface showing Cl⋯H/H⋯Cl inter­actions with red spots indicating close H⋯Cl contacts; white areas match van der Waals radii distances. (*b*) Hirshfeld surface showing H⋯H contacts. (*c*) two-dimensional fingerprint of C⋯H/H⋯C contacts in *d*_norm_ mode. (*d*) Curvedness HS indicating contacts between carbon atoms, showing the π-stacking inter­actions. (*e*) Shape-index plot indicating C⋯Cl/Cl⋯C inter­actions; (*f*) shape-index plot indicating H⋯N/N⋯H inter­actions.

In Fig. 6 ▸*a*, the red spots indicate close H⋯Cl contacts, which can be attributed to the C—H⋯Cl hydrogen bonds. The white and red areas represent regions where the distance between neighboring atoms closely matches the sum of their van der Waals radii, suggesting H⋯Cl contacts. Blue areas indicate instances where neighboring atoms are too distant to inter­act. The 2D FP plot displayed in Fig. 6 ▸*a* illustrates the H⋯Cl/Cl⋯H contacts, which make the most significant contribution to the total Hirshfeld surface area (32.4%). It is characterized by two symmetrical peaks at the top left and bottom right with *d*_e_ + *d*_i_ = 2.7 Å (labeled 1 and 2).

Fig. 6 ▸*b* and 6*c* illustrate the H⋯H contacts and C⋯H/H⋯C contacts respectively, represented by red dots. The 2D FP shown in Fig. 6 ▸*b* shows the two-dimensional (*d*_i_, *d*_e_) points associated with hydrogen atoms (rvdW = 1.20 Å). It features an endpoint towards the origin with *d_i_* = *d*_e_ = 1.1 Å (labeled 3), revealing the presence of close H⋯H contacts, accounting for 30.7% of all inter­molecular contacts. The FP plot in Fig. 6 ▸*c* has symmetrical peaks at the top left and bottom right with *d*_e_ + *d*_i_ = 2.6 Å (labeled 4 and 5), characteristic of C—H⋯π inter­actions (24.0%).

In the HS plotted over curvedness shown in Fig. 6 ▸*d*, the presence of flat regions indicates the existence of π-stacking inter­actions. Fig. 6 ▸*e* and 6*f* illustrate the C⋯Cl/Cl⋯C and N⋯H/H⋯N contacts, respectively. The other contacts shown in the two-dimensional fingerprint plots are C⋯C (6.2%), C⋯Cl/Cl⋯C (4.1%) and N⋯H/H⋯N (1.7%). The minimal contributions of the Cl⋯Cl (0.7%) and N⋯C/C⋯N (0.2%) inter­molecular contacts mean they have a negligible impact on the packing.

## 6. Synthesis and crystallization

To prepare the title compound, a solution of 1,10-phenanthroline (0.090 g, 0.5 mmol) in ethanol (25 ml) and phenyl­tin trichloride (0.151 g, 0.5 mmol) in ethanol (25 ml) was refluxed for 24 h. The white precipitate that formed was removed by filtration. Colorless crystals were obtained after leaving a di­chloro­ethane solution to stand for 7 d at room temperature. Yield: 85%. IR (KBr, cm^−1^): 3054 (Ar—H), 3055 (=C—H), 1628 (C=N), 1430–1627 (C=C), 851 (=C—H), 448 (Sn—C), 423 (Sn—N).

## 7. Refinement

Crystal data, data collection and structure refinement details are summarized in Table 3 ▸. The C-bound H atoms were placed geometrically and refined as riding atoms [C—H = 0.93 Å and *U*_iso_(H) = 1.2*U*_eq_(C)].

## Supplementary Material

Crystal structure: contains datablock(s) import, I. DOI: 10.1107/S2056989024009150/zn2038sup1.cif

Structure factors: contains datablock(s) I. DOI: 10.1107/S2056989024009150/zn2038Isup2.hkl

FT-IR Spectra (figure S1). DOI: 10.1107/S2056989024009150/zn2038sup3.png

CCDC reference: 2370790

Additional supporting information:  crystallographic information; 3D view; checkCIF report

## Figures and Tables

**Table 1:** Selected geometric parameters (Å, °)

Sn1—Cl3	2.4530 (6)	Sn1—N2	2.2728 (19)
Sn1—Cl1	2.4419 (6)	Sn1—N1	2.2802 (19)
Sn1—Cl2	2.4067 (6)	Sn1—C1	2.145 (2)
			
Cl1—Sn1—Cl3	165.07 (2)	N2—Sn1—N1	72.77 (7)
Cl2—Sn1—Cl3	91.82 (2)	N1—Sn1—Cl3	85.10 (5)
Cl2—Sn1—Cl1	93.08 (2)	N1—Sn1—Cl1	86.12 (5)
N2—Sn1—Cl3	82.62 (5)	N1—Sn1—Cl2	163.04 (5)
N2—Sn1—Cl1	83.27 (5)	C1—Sn1—N2	168.92 (8)
N2—Sn1—Cl2	90.30 (5)		

**Table 2:** Hydrogen-bond geometry (Å, °)

*D*—H⋯*A*	*D*—H	H⋯*A*	*D*⋯*A*	*D*—H⋯*A*
C15—H15⋯Cl3^i^	0.95	2.94	3.851 (1)	139
C5—H5⋯Cl3^ii^	0.95	2.84	3.773 (2)	166
C9—H9⋯Cl1^iii^	0.95	2.87	3.683 (2)	144
C2—H2⋯Cl1	0.95	2.75	3.392 (2)	126
C6—H6⋯Cl3	0.95	2.86	3.411 (1)	124
C16—H16⋯Cl2	0.95	2.97	3.328 (3)	126
C7—H7⋯Cl2^iv^	0.95	2.85	3.654 (1)	143
C12—H12⋯Cl2^v^	0.95	2.97	3.693 (4)	133

Symmetry codes: (i) 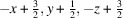; (ii) 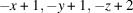; (iii) 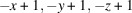; (iv) 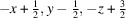; (v) 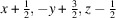.

**Table 3:** Experimental details

Crystal data	
Chemical formula	[Sn(C_6_H_5_)Cl_3_(C_12_H_8_N_2_)]
*M*_r_	482.34
Crystal system, space group	Monoclinic, *P*2_1_/*n*
Temperature (K)	150
*a*, *b*, *c* (Å)	9.1085 (9), 13.1958 (13), 14.9869 (14)
β (°)	102.261 (3)
*V* (Å^3^)	1760.2 (3)
*Z*	4
Radiation type	Mo *K*α
μ (mm^−1^)	1.91
Crystal size (mm)	0.4 × 0.3 × 0.2

Data collection	
Diffractometer	Bruker D8 VENTURE
Absorption correction	Multi-scan (*SADABS*; Krause et al., 2015 ▸)
No. of measured, independent and observed [*I* > 2σ(*I*)] reflections	23358, 4356, 3974
*R*_int_	0.033
(sin θ/λ)_max_ (Å^−1^)	0.667

Refinement	
*R*[*F*^2^ > 2σ(*F*^2^)], *wR*(*F*^2^), *S*	0.025, 0.055, 1.14
No. of reflections	3974
No. of parameters	217
H-atom treatment	H-atom parameters constrained
Δρ_max_, Δρ_min_ (e Å^−3^)	0.44, −0.77

Computer programs: *APEX4* and *SAINT* (Bruker, 2014 ▸), *SHELXT2018/2* (Sheldrick, 2015*a* ▸), *SHELXL2018/3* (Sheldrick, 2015*b* ▸) and *OLEX2* (Dolomanov *et al.*, 2009 ▸).

## supplementary crystallographic information

### Trichlorido(1,10-phenanthroline-κ2N,N')phenyltin(IV) Crystal data

**Table:**

[Sn(C_6_H_5_)Cl_3_(C_12_H_8_N_2_)]	*F*(000) = 944
*M**_r_* = 482.34	*D*_x_ = 1.820 Mg m^−^^3^
Monoclinic, *P*2_1_/*n*	Mo *K*α radiation, λ = 0.71073 Å
*a* = 9.1085 (9) Å	Cell parameters from 9933 reflections
*b* = 13.1958 (13) Å	θ = 2.6–27.5°
*c* = 14.9869 (14) Å	µ = 1.91 mm^−^^1^
β = 102.261 (3)°	*T* = 150 K
*V* = 1760.2 (3) Å^3^	Prism, clear yellowish colourless
*Z* = 4	0.4 × 0.3 × 0.2 mm

### Trichlorido(1,10-phenanthroline-κ2N,N')phenyltin(IV) Data collection

**Table:**

Bruker D8 VENTURE diffractometer	3974 reflections with *I* > 2σ(*I*)
rotation images scans	*R*_int_ = 0.033
Absorption correction: multi-scan (SADABS; Krause et al., 2015)	θ_max_ = 28.3°, θ_min_ = 2.4°
	*h* = −12→12
23358 measured reflections	*k* = −17→17
4356 independent reflections	*l* = −19→18

### Trichlorido(1,10-phenanthroline-κ2N,N')phenyltin(IV) Refinement

**Table:**

Refinement on *F*^2^	Primary atom site location: dual
Least-squares matrix: full	Hydrogen site location: inferred from neighbouring sites
*R*[*F*^2^ > 2σ(*F*^2^)] = 0.025	H-atom parameters constrained
*wR*(*F*^2^) = 0.055	*w* = 1/[σ^2^(*F*_o_^2^) + (0.0068*P*)^2^ + 2.7739*P*] where *P* = (*F*_o_^2^ + 2*F*_c_^2^)/3
*S* = 1.14	(Δ/σ)_max_ = 0.001
3974 reflections	Δρ_max_ = 0.44 e Å^−^^3^
217 parameters	Δρ_min_ = −0.77 e Å^−^^3^
0 restraints	

### Trichlorido(1,10-phenanthroline-κ2N,N')phenyltin(IV) Special details

**Table:**

Geometry. All esds (except the esd in the dihedral angle between two l.s. planes) are estimated using the full covariance matrix. The cell esds are taken into account individually in the estimation of esds in distances, angles and torsion angles; correlations between esds in cell parameters are only used when they are defined by crystal symmetry. An approximate (isotropic) treatment of cell esds is used for estimating esds involving l.s. planes.
Refinement. Data collection was done using MoKα radiation with a D8 VENTURE Bruker-AXS diffractometer. Cell refinement and data reduction were performed using the SAINT program. The structure was solved using Olex2 (Dolomanov *et al.*, 2009) and the SHELXT (Sheldrick, 2018) program with intrinsic phasing. It was refined with the SHELXL (Sheldrick, 2015) package using least squares minimization.

### Trichlorido(1,10-phenanthroline-κ2N,N')phenyltin(IV) Fractional atomic coordinates and isotropic or equivalent isotropic displacement parameters (Å^2^)

**Table:**

	*x*	*y*	*z*	*U*_iso_*/*U*_eq_	
Sn1	0.43722 (2)	0.69190 (2)	0.74892 (2)	0.01266 (5)	
Cl3	0.65056 (6)	0.64521 (5)	0.87156 (4)	0.02106 (12)	
Cl1	0.27434 (6)	0.73650 (4)	0.60281 (4)	0.01988 (11)	
Cl2	0.42982 (7)	0.86043 (4)	0.80920 (4)	0.02232 (12)	
N2	0.6229 (2)	0.73932 (15)	0.67755 (13)	0.0159 (4)	
N1	0.5013 (2)	0.55247 (14)	0.67579 (13)	0.0152 (4)	
C1	0.2682 (2)	0.61864 (17)	0.80618 (15)	0.0150 (4)	
C17	0.6782 (2)	0.66479 (18)	0.63117 (15)	0.0161 (4)	
C18	0.6156 (2)	0.56548 (18)	0.63148 (15)	0.0159 (4)	
C7	0.4377 (3)	0.46156 (18)	0.67603 (16)	0.0201 (5)	
H7	0.356976	0.453027	0.706415	0.024*	
C2	0.1193 (3)	0.64428 (19)	0.77293 (17)	0.0212 (5)	
H2	0.093114	0.687743	0.721470	0.025*	
C3	0.0071 (3)	0.6065 (2)	0.81476 (18)	0.0263 (5)	
H3	−0.094731	0.625359	0.792408	0.032*	
C10	0.6716 (3)	0.48507 (19)	0.58645 (16)	0.0195 (5)	
C8	0.4872 (3)	0.37828 (19)	0.63242 (17)	0.0247 (5)	
H8	0.439814	0.314245	0.633125	0.030*	
C16	0.6795 (3)	0.83217 (19)	0.67801 (17)	0.0218 (5)	
H16	0.641490	0.884058	0.710796	0.026*	
C13	0.7940 (2)	0.6826 (2)	0.58379 (16)	0.0199 (5)	
C6	0.3047 (3)	0.55157 (19)	0.87977 (16)	0.0209 (5)	
H6	0.406433	0.532876	0.902577	0.025*	
C15	0.7934 (3)	0.8560 (2)	0.63175 (18)	0.0265 (5)	
H15	0.831083	0.923282	0.633077	0.032*	
C5	0.1922 (3)	0.5121 (2)	0.91977 (17)	0.0246 (5)	
H5	0.216871	0.464759	0.968438	0.030*	
C11	0.7900 (3)	0.5055 (2)	0.53918 (17)	0.0253 (5)	
H11	0.828444	0.451887	0.508489	0.030*	
C4	0.0444 (3)	0.5419 (2)	0.88845 (17)	0.0256 (5)	
H4	−0.031568	0.517732	0.917810	0.031*	
C9	0.6043 (3)	0.38929 (19)	0.58869 (17)	0.0249 (5)	
H9	0.639571	0.332705	0.560140	0.030*	
C12	0.8478 (3)	0.5997 (2)	0.53763 (17)	0.0259 (6)	
H12	0.925432	0.611268	0.505372	0.031*	
C14	0.8503 (3)	0.7819 (2)	0.58447 (18)	0.0261 (5)	
H14	0.927037	0.797456	0.552514	0.031*	

### Trichlorido(1,10-phenanthroline-κ2N,N')phenyltin(IV) Atomic displacement parameters (Å^2^)

**Table:**

	*U*^11^	*U*^22^	*U*^33^	*U*^12^	*U*^13^	*U*^23^
Sn1	0.01200 (7)	0.01385 (8)	0.01264 (8)	0.00132 (5)	0.00377 (5)	−0.00003 (5)
Cl3	0.0157 (2)	0.0260 (3)	0.0195 (3)	0.0018 (2)	−0.0009 (2)	0.0031 (2)
Cl1	0.0206 (3)	0.0237 (3)	0.0144 (2)	0.0032 (2)	0.0017 (2)	0.0026 (2)
Cl2	0.0275 (3)	0.0174 (3)	0.0234 (3)	0.0021 (2)	0.0084 (2)	−0.0055 (2)
N2	0.0141 (9)	0.0171 (9)	0.0163 (9)	0.0006 (7)	0.0030 (7)	0.0007 (7)
N1	0.0159 (9)	0.0154 (9)	0.0149 (9)	0.0003 (7)	0.0045 (7)	−0.0011 (7)
C1	0.0152 (10)	0.0154 (10)	0.0150 (10)	−0.0009 (8)	0.0047 (8)	−0.0013 (8)
C17	0.0140 (10)	0.0211 (11)	0.0129 (10)	0.0018 (8)	0.0023 (8)	0.0012 (8)
C18	0.0151 (10)	0.0201 (11)	0.0125 (10)	0.0042 (8)	0.0029 (8)	0.0004 (8)
C7	0.0210 (11)	0.0177 (11)	0.0215 (12)	−0.0017 (9)	0.0041 (9)	0.0006 (9)
C2	0.0163 (11)	0.0258 (13)	0.0219 (12)	0.0025 (9)	0.0048 (9)	0.0037 (10)
C3	0.0150 (11)	0.0359 (15)	0.0301 (14)	−0.0020 (10)	0.0092 (10)	0.0010 (11)
C10	0.0199 (11)	0.0225 (12)	0.0149 (11)	0.0089 (9)	0.0012 (9)	−0.0015 (9)
C8	0.0323 (14)	0.0166 (11)	0.0238 (12)	0.0008 (10)	0.0027 (10)	−0.0016 (9)
C16	0.0204 (11)	0.0194 (12)	0.0245 (12)	−0.0003 (9)	0.0026 (9)	0.0030 (9)
C13	0.0123 (10)	0.0324 (13)	0.0153 (11)	0.0037 (9)	0.0035 (8)	0.0064 (9)
C6	0.0218 (11)	0.0234 (12)	0.0176 (11)	0.0031 (9)	0.0044 (9)	0.0028 (9)
C15	0.0242 (12)	0.0261 (13)	0.0287 (14)	−0.0061 (10)	0.0046 (10)	0.0091 (11)
C5	0.0320 (13)	0.0254 (13)	0.0171 (11)	−0.0062 (10)	0.0069 (10)	0.0053 (10)
C11	0.0217 (12)	0.0361 (15)	0.0192 (12)	0.0135 (11)	0.0071 (9)	−0.0016 (10)
C4	0.0262 (13)	0.0345 (14)	0.0195 (12)	−0.0106 (11)	0.0120 (10)	−0.0030 (10)
C9	0.0312 (13)	0.0212 (12)	0.0203 (12)	0.0118 (10)	0.0008 (10)	−0.0035 (9)
C12	0.0171 (11)	0.0440 (16)	0.0187 (12)	0.0082 (11)	0.0086 (9)	0.0027 (11)
C14	0.0165 (11)	0.0380 (15)	0.0244 (13)	−0.0031 (10)	0.0058 (9)	0.0114 (11)

### Trichlorido(1,10-phenanthroline-κ2N,N')phenyltin(IV) Geometric parameters (Å, º)

**Table:**

Sn1—Cl3	2.4530 (6)	C10—C11	1.436 (3)
Sn1—Cl1	2.4419 (6)	C10—C9	1.409 (4)
Sn1—Cl2	2.4067 (6)	C8—H8	0.9500
Sn1—N2	2.2728 (19)	C8—C9	1.373 (4)
Sn1—N1	2.2802 (19)	C16—H16	0.9500
Sn1—C1	2.145 (2)	C16—C15	1.400 (3)
N2—C17	1.361 (3)	C13—C12	1.434 (4)
N2—C16	1.329 (3)	C13—C14	1.406 (4)
N1—C18	1.359 (3)	C6—H6	0.9500
N1—C7	1.333 (3)	C6—C5	1.393 (3)
C1—C2	1.384 (3)	C15—H15	0.9500
C1—C6	1.398 (3)	C15—C14	1.373 (4)
C17—C18	1.429 (3)	C5—H5	0.9500
C17—C13	1.411 (3)	C5—C4	1.386 (4)
C18—C10	1.410 (3)	C11—H11	0.9500
C7—H7	0.9500	C11—C12	1.352 (4)
C7—C8	1.401 (3)	C4—H4	0.9500
C2—H2	0.9500	C9—H9	0.9500
C2—C3	1.399 (3)	C12—H12	0.9500
C3—H3	0.9500	C14—H14	0.9500
C3—C4	1.379 (4)		
			
Cl1—Sn1—Cl3	165.07 (2)	C4—C3—H3	120.0
Cl2—Sn1—Cl3	91.82 (2)	C18—C10—C11	118.8 (2)
Cl2—Sn1—Cl1	93.08 (2)	C9—C10—C18	117.4 (2)
N2—Sn1—Cl3	82.62 (5)	C9—C10—C11	123.8 (2)
N2—Sn1—Cl1	83.27 (5)	C7—C8—H8	120.0
N2—Sn1—Cl2	90.30 (5)	C9—C8—C7	120.0 (2)
N2—Sn1—N1	72.77 (7)	C9—C8—H8	120.0
N1—Sn1—Cl3	85.10 (5)	N2—C16—H16	118.9
N1—Sn1—Cl1	86.12 (5)	N2—C16—C15	122.2 (2)
N1—Sn1—Cl2	163.04 (5)	C15—C16—H16	118.9
C1—Sn1—Cl3	96.23 (6)	C17—C13—C12	119.0 (2)
C1—Sn1—Cl1	96.68 (6)	C14—C13—C17	117.5 (2)
C1—Sn1—Cl2	100.76 (6)	C14—C13—C12	123.5 (2)
C1—Sn1—N2	168.92 (8)	C1—C6—H6	119.9
C1—Sn1—N1	96.16 (8)	C5—C6—C1	120.2 (2)
C17—N2—Sn1	115.73 (15)	C5—C6—H6	119.9
C16—N2—Sn1	125.32 (16)	C16—C15—H15	120.1
C16—N2—C17	118.9 (2)	C14—C15—C16	119.7 (2)
C18—N1—Sn1	115.71 (15)	C14—C15—H15	120.1
C7—N1—Sn1	124.69 (15)	C6—C5—H5	120.1
C7—N1—C18	119.6 (2)	C4—C5—C6	119.9 (2)
C2—C1—Sn1	118.52 (17)	C4—C5—H5	120.1
C2—C1—C6	119.3 (2)	C10—C11—H11	119.4
C6—C1—Sn1	122.04 (17)	C12—C11—C10	121.1 (2)
N2—C17—C18	118.0 (2)	C12—C11—H11	119.4
N2—C17—C13	122.3 (2)	C3—C4—C5	120.2 (2)
C13—C17—C18	119.7 (2)	C3—C4—H4	119.9
N1—C18—C17	117.7 (2)	C5—C4—H4	119.9
N1—C18—C10	122.1 (2)	C10—C9—H9	120.2
C10—C18—C17	120.2 (2)	C8—C9—C10	119.5 (2)
N1—C7—H7	119.3	C8—C9—H9	120.2
N1—C7—C8	121.4 (2)	C13—C12—H12	119.4
C8—C7—H7	119.3	C11—C12—C13	121.2 (2)
C1—C2—H2	119.8	C11—C12—H12	119.4
C1—C2—C3	120.3 (2)	C13—C14—H14	120.3
C3—C2—H2	119.8	C15—C14—C13	119.4 (2)
C2—C3—H3	120.0	C15—C14—H14	120.3
C4—C3—C2	120.0 (2)		
			
Sn1—N2—C17—C18	0.0 (3)	C18—N1—C7—C8	0.8 (3)
Sn1—N2—C17—C13	−179.75 (16)	C18—C17—C13—C12	−0.7 (3)
Sn1—N2—C16—C15	−179.48 (18)	C18—C17—C13—C14	179.1 (2)
Sn1—N1—C18—C17	−3.1 (3)	C18—C10—C11—C12	0.1 (4)
Sn1—N1—C18—C10	177.34 (17)	C18—C10—C9—C8	1.1 (3)
Sn1—N1—C7—C8	−177.42 (18)	C7—N1—C18—C17	178.6 (2)
Sn1—C1—C2—C3	−173.21 (19)	C7—N1—C18—C10	−1.0 (3)
Sn1—C1—C6—C5	174.69 (18)	C7—C8—C9—C10	−1.3 (4)
N2—C17—C18—N1	2.1 (3)	C2—C1—C6—C5	−0.8 (4)
N2—C17—C18—C10	−178.3 (2)	C2—C3—C4—C5	−1.7 (4)
N2—C17—C13—C12	179.1 (2)	C10—C11—C12—C13	0.6 (4)
N2—C17—C13—C14	−1.1 (3)	C16—N2—C17—C18	−179.9 (2)
N2—C16—C15—C14	−0.4 (4)	C16—N2—C17—C13	0.3 (3)
N1—C18—C10—C11	178.4 (2)	C16—C15—C14—C13	−0.5 (4)
N1—C18—C10—C9	0.1 (3)	C13—C17—C18—N1	−178.2 (2)
N1—C7—C8—C9	0.4 (4)	C13—C17—C18—C10	1.4 (3)
C1—C2—C3—C4	−1.2 (4)	C6—C1—C2—C3	2.4 (4)
C1—C6—C5—C4	−2.1 (4)	C6—C5—C4—C3	3.3 (4)
C17—N2—C16—C15	0.5 (3)	C11—C10—C9—C8	−177.2 (2)
C17—C18—C10—C11	−1.2 (3)	C9—C10—C11—C12	178.4 (2)
C17—C18—C10—C9	−179.5 (2)	C12—C13—C14—C15	−179.0 (2)
C17—C13—C12—C11	−0.3 (4)	C14—C13—C12—C11	179.9 (2)
C17—C13—C14—C15	1.2 (4)		

### Trichlorido(1,10-phenanthroline-κ2N,N')phenyltin(IV) Hydrogen-bond geometry (Å, º)

**Table:**

*D*—H···*A*	*D*—H	H···*A*	*D*···*A*	*D*—H···*A*
C15—H15···Cl3^i^	0.95	2.94	3.851 (1)	139
C5—H5···Cl3^ii^	0.95	2.84	3.773 (2)	166
C9—H9···Cl1^iii^	0.95	2.87	3.683 (2)	144
C2—H2···Cl1	0.95	2.75	3.392 (2)	126
C6—H6···Cl3	0.95	2.86	3.411 (1)	124
C16—H16···Cl2	0.95	2.97	3.328 (3)	126
C7—H7···Cl2^iv^	0.95	2.85	3.654 (1)	143
C12—H12···Cl2^v^	0.95	2.97	3.693 (4)	133

Symmetry codes: (i) −*x*+3/2, *y*+1/2, −*z*+3/2; (ii) −*x*+1, −*y*+1, −*z*+2; (iii) −*x*+1, −*y*+1, −*z*+1; (iv) −*x*+1/2, *y*−1/2, −*z*+3/2; (v) *x*+1/2, −*y*+3/2, *z*−1/2.
