# Supplementary figures and images for: Crystal structure and Hirshfeld surface analysis of tri­chlorido­(1,10-phenanthroline-κ2N,N′)phenyltin(IV)
Source: Acta Crystallogr E Crystallogr Commun. 2024 Sep 24;80(Pt 10):1039–43. doi: 10.1107/S2056989024009150 (PMC11451494; doi:10.1107/S2056989024009150)

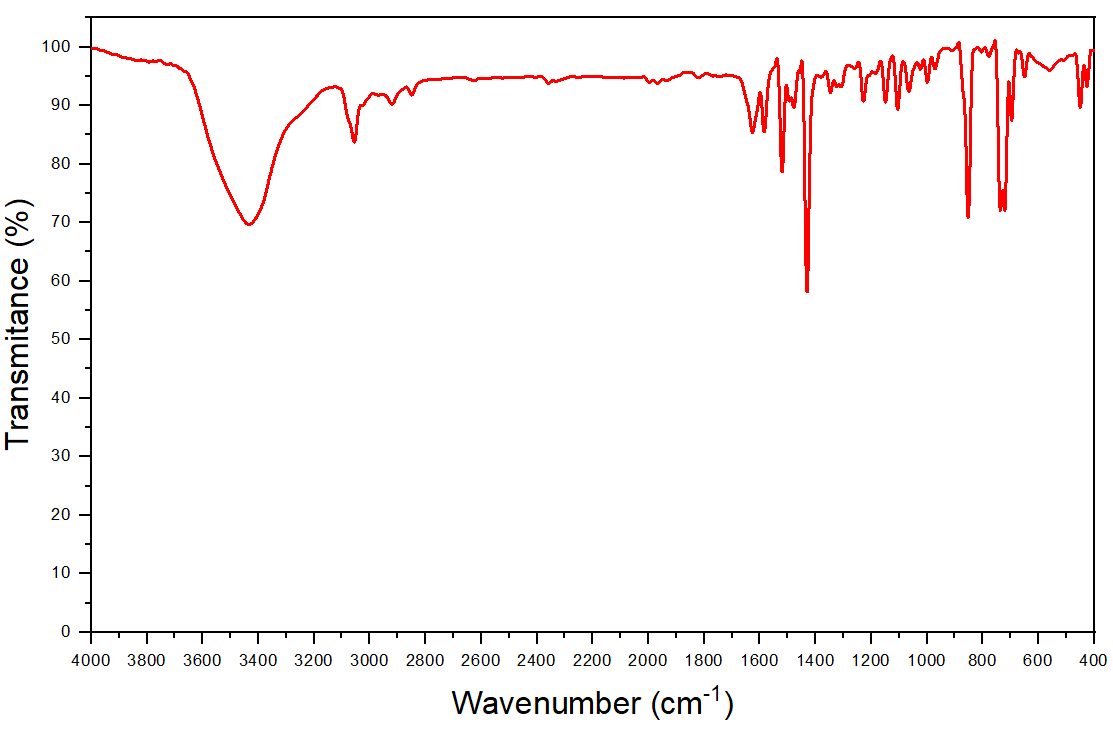

Supplement: Supplementary file 3 [file e-80-01039-sup3.png]
